# Comparison of vision-related quality of life and mental health between congenital and acquired low-vision patients

**DOI:** 10.1038/s41433-019-0439-6

**Published:** 2019-04-24

**Authors:** Sang Uk Choi, Yeoun Sook Chun, Jeong Kyu Lee, Jee Taek Kim, Jae Hoon Jeong, Nam Ju Moon

**Affiliations:** 1 Department of Ophthalmology, College of Medicine, Chung-Ang University Hospital, Seoul, Republic of Korea; 2 Departement of Ophthalmology, Konyang University Hospital, Daejeon, Republic of Korea

**Keywords:** Rehabilitation, Outcomes research

## Abstract

**Purpose:**

To evaluate the impact of the age of onset of low vision on patients’ vision-related quality of life (VR-QoL) and mental health.

**Methods:**

Low-vision patients who visited Chung-Ang University hospital from January 2012 to December 2014 were included. Patients were divided into the congenital low-vision (CLV) and acquired low-vision (ALV) groups according to the age of disease onset. People with normal visual function comprised the control group. VR-QoL was estimated with the National Eye Institute Visual Function Questionnaire (NEI VFQ-25), while mental health was assessed through the Beck Depression Inventory (BDI) and the Beck Anxiety Inventory (BAI). The mean scores of each questionnaire were compared between the groups in independent *t*-tests.

**Results:**

Overall, 125 low-vision patients (55 CLV and 70 ALV) and 71 control subjects were included. Although the subscale and composite scores of the NEI VFQ-25 were lower in the ALV group than in the CLV group, the differences were not significant. However, the BDI and BAI scores were significantly higher in the ALV group than in the CLV group (12.07 ± 11.97 vs. 7.67 ± 9.04, *P* = 0.021; 9.11 ± 10.51 vs. 5.69 ± 6.85, *P* = 0.030, respectively). Also, the number of patients requiring expert consultation for depression was higher in the ALV group than in the CLV group (*P* = 0.010).

**Conclusion:**

ALV patients have more vulnerable mental health states than CLV patients. Therefore, assessment of the age of onset of low vision and mental health plays a critical role in successful rehabilitation.

## Introduction

Low vision has been declared by the World Health Organization (WHO) to be one of the major ophthalmologic problems requiring global attention [1]. According to global data from the WHO on visual impairments, preventable infectious causes of visual impairment (e.g., trachoma and corneal opacities) have been declining, while posterior segment diseases have become increasingly important causes of visual impairment due to the rapid growth of the aging population [2, 3]. In fact, between 1990 and 2010, the number of low-vision patients with macular disease increased dramatically by 81% (2.7 million) [4], and the global number of people with low vision increased by 19 million (from 172 million) in the same time period [5]. These statistics highlight the increase in late-onset low vision and the urgent need for eye care systems that address chronic eye diseases with rehabilitation and support services.

Low-vision patients experience a reduced vision-related quality of life (VR-QoL) due to impaired visual function [6]. Therefore, the final goal of low-vision rehabilitation is to improve the daily quality of life [7]. Accordingly, objective assessment of VR-QoL in low-vision patients is an important part of the rehabilitation process. Likewise, low vision has been associated with lower psychosocial wellbeing, manifested as a loss of interest in and enjoyment of physical activities [8]. Reduced psychosocial wellbeing is expressed as an adverse mental health status, including feelings of social isolation, depression, and anxiety [9–11]. Impaired mental health can impact low-vision rehabilitation and even cause its failure [12, 13]. In particular, in cases of acquired low vision (ALV), even mild deterioration of visual function can seriously impact patient VR-QoL and mental health [13].

In low-vision rehabilitation, visual acuity and visual field tests are objective methods of evaluating visual function. However, such objective examinations cannot be used to measure subjective parameters, such as VR-QoL and mental health. Also, the impact of the age of onset of low vision on patients’ VR-QoL and mental health has not been studied previously. Thus, the purpose of this study was to evaluate VR-QoL and mental health in low-vision patients and determine the impact of the age of onset by comparing VR-QoL and mental health in patients with congenital low vision (CLV) and ALV.

## Methods

### Study subjects

Low-vision patients (*N* = 125) who visited the low-vision clinic of Chung-Ang University hospital from January 2012 to December 2014 were included. After receiving a low vision diagnosis at another medical institution, the previous medical records were reviewed and recorded when the patient was transferred to our institution. Patients were excluded if they were less than 18 years old, could not understand the questionnaire due to an intellectual disability, had a history of psychiatric treatment due to a depressive/anxiety disorder, or had cerebrovascular disease. The questionnaire was filled out by the interviewers when it was difficult for the patient to self-report due to the visual impairment. In the same period, 71 people with normal visual function and no ophthalmic disease enrolled in the control group and completed the same self-report questionnaire. The protocol was reviewed and approved by the Institutional Review Board and Ethics Committee of Chung-Ang University, Seoul, Republic of Korea. The methods applied in the study adhered to the tenets of the declaration of Helsinki. The informed consent was obtained from the all of the subjects.

The CLV group was defined as the patients who were diagnosed with low vision before 5 years of age. Patients who were diagnosed after 5 years of age were defined as the ALV group. A Snellen chart (Precision Vision, La Salle, IL, USA) was used to measure the distance visual acuity (VA) for each eye at a distance of 5 m. If the VA was lower than 6/60, a Feinbloom chart (Precision Vision) or a Low-Vision Letter chart (Precision Vision) was used. The near VA was measured from 40 cm with a Lighthouse Near Visual Acuity Test Chart (Lighthouse International Incorporated, New York, NY, USA) under light from 600 to 700 lux. The participant’s usual distance correction was initially used in the assessment of low vision. Participants were asked to read the numbers on the VA chart while the forced choice method was consistently applied. The VA was recorded as the smallest line in which participants could correctly read more than 60% of the numbers. The best corrected visual acuity (BCVA) was obtained from the VA examination performed with full subjective refraction. In accordance with the criteria of the International Classification of Diseases (tenth revision), the patients whose VA was worse than 6/18 and equal to or better than 6/60 were classified as having moderate visual impairment, and those whose VA was worse than 6/60 and equal to or better than 3/60 were classified as having severe visual impairment. The time between the initial visit to the low-vision clinic for rehabilitation and the study enrollment was set as the rehabilitation period.

### Questionnaire analysis

The National Eye Institute Visual Function Questionnaire (NEI VFQ-25) consists of 25 items and 14 additional items comparable to 25 items that used to verify reliability of the answer of 25 items questionnaire. The 25 items are divided into 12 subscales. Each item is scored from 0 to 100, where a higher score indicates a better state of wellbeing for the subscale of that item. The driving subscale was not used in the questionnaire because none of the low-vision patients were able to drive. The mean scores for the 12 subscales and the total composite scores were compared between the groups.

The Beck Depression Inventory (BDI), designed by Beck et al., is commonly used to screen for depressive disorder [14] and is considered to be a representative psychiatric interview [15]. The questionnaire is composed of 21 items, each of which is scored from 0 to 3, such that the total score of the questionnaire ranges from 0 to 63. A higher score for an item indicates worse depression in daily life. In accordance with the study of Rhee et al., we used the following cutoff values: in men, (1) <9 normal, (2) 10–15 mild depressive state, (3) 16–19 moderate depressive state, (4) 20–23 mild depressive disorder, and (5) ≥24 severe depressive disorder; in women, (1) <9 normal, (2) 10–16 mild depressive state, (3) 17–20 moderate depressive state, (4) 21–24 mild depressive disorder, and (5) ≥25 severe depressive disorder [16]. Therefore, men with scores greater than or equal to 16 and women with scores greater than or equal to 17 were regarded as needing an expert consultation for an evaluation of the depressed state.

The Beck Anxiety Inventory (BAI), which evaluates anxiety symptoms in daily life, was translated to Korean by Seo in 1996 [17]. It is composed of 21 items, each of which is scored from 0 to 3, such that the total score of the questionnaire ranges from 0 to 63. A higher score for an item indicates worse anxiety in daily life. In accordance with a previous study, we used the following cutoff values: (1) 22–26 mild anxiety state, (2) 27–31 moderate anxiety state, (3) 27–31 severe anxiety state, and (4) ≥32 very severe anxiety state. Therefore, patients with scores greater than or equal to 22 were regarded as needing an expert consultation for an evaluation of the anxiety.

### Statistical analysis

Demographic characteristics are represented as means with standard deviations as appropriate. Independent *t*-tests were performed to compare the VR-QoL, depression, and anxiety scores between the low-vision and control groups, and between the CLV and ALV groups. To control the confounding effect of demographic data, the analysis of covariance (ANCOVA) was performed, if there are demographic factors with significant differences. Additionally, correlation coefficients were calculated so that the correlation of the rehabilitation period with the NEI VFQ-25 composite score, the BDI score and the BAI score could be determined. All *P* values were two-sided, and differences were considered statistically significant when the *P* values were less than 0.05. All statistical analyses were performed with SPSS 20.0 (SPSS ver. 20 for Windows; SPSS Incorporated, Chicago, IL, USA).

## Results

The demographic data of all the study groups are summarized in Table 1. In the low-vision group, there were no significant differences between the CLV and ALV groups in sex or area of residence; however, the CLV group was younger than the ALV group (*P* < 0.001). The degree of visual impairment did not differ significantly between the groups (*P* = 0.171). The CLV group had a longer rehabilitation period than the ALV group (*P* = 0.009). The principal causes visual impairment in the CLV and ALV groups were optic atrophy (32.7 and 20.0%, respectively) and macular dystrophy and degeneration (16.4 and 28.6%, respectively) (Supplementary table 1).

**Table 1:** Demographic characteristics of the low-vision (congenital and acquired low-vision) groups and the control group

Variables	Low-vision group				Control (*n* = 71)	*P* value**
	Congenital (*n* = 55) (%)	Acquired (*n* = 70) (%)	*P* value*	Total (*n* = 125) (%)		
Sex						
Male	24 (43.63)	42 (60.00)	0.069^a^	66 (52.80)	39 (54.93)	0.774^a^
Female	31 (56.37)	28 (40.00)		59 (47.20)	32 (45.07)	
Age (years)	31.89 ± 13.31	45.47 ± 14.42	<0.001^b^	39.50 ± 15.45	37.55 ± 14.22	0.395^b^
Area of residence						
Rural	29 (52.73)	27 (38.57)	0.114^a^	56 (44.80)	40 (56.34)	0.120^a^
Urban	26 (47.27)	43 (61.43)		69 (55.20)	31 (43.66)	
Degree of visual impairment						
Moderate visual impairment (6/18 > VA ≥ 6/60)	35 (63.64)	36 (51.43)	0.171^a^	71 (56.80)	0	N/A
Severe visual impairment (6/60 > VA ≥ 3/60)	20 (36.36)	34 (48.57)		54 (43.20)	0	N/A
Duration of rehabilitation (years)	10.11 ± 7.75	6.73 ± 6.49	0.009^b^	8.21 ± 7.24	N/A	N/A

*Comparison between congenital group and acquired group; *P* value < 0.05, statistically significant

**Comparison between low-vision group and control group; *P* value < 0.05, statistically significant

^a^Chi-square test.

^b^Independent *t*-test

The mean NEI VFQ-25 subscale and composite scores were significantly lower in the low-vision group than in the normal control group (Table 2), and were generally lower in the ALV group than in the CLV group. However, the differences between the ALV and CLV groups were not significant. On the other hand, the patients with severe visual impairment had significantly lower NEI VFQ-25 subscale and composite scores (except on the general health and ocular pain subscales) than those with moderate visual impairment (Table 3).

**Table 2:** The mean National Eye Institute Visual Function Questionnaire (NEI VFQ-25) subscale and composite scores compared between the congenital low-vision and acquired low-vision groups, and between the low-vision and control groups

	Low vision				Control (*n* = 71)	*P* value**
	Congenital (*n* = 55)	Acquired (*n* = 70)	*P* value*	Total (*n* = 125)		
General health	49.55 ± 25.68	49.64 ± 29.33	0.985	49.60 ± 27.67	67.05 ± 29.79	<0.001
General vision	41.82 ± 26.18	38.29 ± 24.73	0.441	39.84 ± 25.34	78.18 ± 14.77	<0.001
Ocular pain	71.59 ± 27.16	64.29 ± 27.61	0.142	67.50 ± 27.54	92.61 ± 8.19	<0.001
Near vision	47.88 ± 31.31	43.57 ± 32.17	0.454	45.47 ± 31.74	97.35 ± 5.30	<0.001
Distance vision	48.33 ± 29.89	42.14 ± 30.79	0.261	44.87 ± 30.43	90.91 ± 11.85	<0.001
Social functioning	55.52 ± 32.16	48.75 ± 33.99	0.260	51.73 ± 30.43	97.73 ± 4.86	<0.001
Mental health	58.18 ± 28.64	53.93 ± 29.34	0.418	55.80 ± 28.99	96.31 ± 4.09	<0.001
Role difficulties	55.00 ± 36.53	45.18 ± 32.01	0.112	49.50 ± 34.28	96.59 ± 7.76	<0.001
Dependence	57.94 ± 31.56	56.43 ± 32.54	0.794	57.09 ± 31.99	97.64 ± 4.43	<0.001
Driving	N/A	N/A	N/A	N/A	N/A	N/A
Color vision	66.82 ± 31.20	55.36 ± 35.07	0.059	60.40 ± 33.78	98.45 ± 3.21	<0.001
Peripheral vision	53.18 ± 34.04	51.43 ± 32.38	0.769	52.20 ± 32.99	95.45 ± 9.71	<0.001
Composite score	55.37 ± 24.34	50.11 ± 24.43	0.234	52.43 ± 24.43	92.02 ± 6.42	<0.001

Data are presented as mean ± standard deviation

*Comparison between the congenital group and the acquired group; independent *t*-test *P* value < 0.05, statistically significant

**Comparison between the low-vision group and the control group; independent *t*-test *P* value < 0.05, statistically significant

**Table 3:** The mean National Eye Institute Visual Function Questionnaire (NEI VFQ-25) subscale and composite scores in the low-vision group compared by the degree of visual impairment

	Moderate visual impairment (*n* = 71)	Severe visual impairment (*n* = 54)	*P* value*
General health	53.52 ± 27.16	44.44 ± 27.76	0.069
General vision	44.51 ± 26.45	33.70 ± 22.59	0.018
Ocular pain	69.54 ± 26.03	64.81 ± 29.45	0.344
Near vision	54.23 ± 31.96	33.95 ± 27.75	<0.001
Distance vision	54.34 ± 28.62	32.41 ± 28.40	<0.001
Social functioning	59.73 ± 30.69	41.20 ± 33.79	0.002
Mental health	60.92 ± 28.64	49.07 ± 28.33	0.023
Role difficulties	55.63 ± 33.79	41.44 ± 33.54	0.021
Dependency	62.37 ± 32.96	50.15 ± 29.56	0.034
Driving	N/A	N/A	N/A
Color vision	68.31 ± 31.61	50.00 ± 33.99	0.002
Peripheral vision	60.92 ± 30.09	40.74 ± 33.39	0.001
Composite score	58.59 ± 24.59	44.32 ± 21.89	0.001

Data are presented as means ± standard deviations

*Independent *t*-test was performed; *P* value < 0.05, statistically significant

The mean BDI and BAI scores were significantly lower in the CLV group than in the ALV group (7.67 ± 9.04 and 12.07 ± 11.97, respectively, for the BDI, *P* = 0.021; 5.69 ± 6.85; and 9.11 ± 10.52, respectively, for the BAI, *P* = 0.030) (Table 4). Also, 27 (21.6%) and 9 (7.2%) patients with low vision required expert consultation for depression and anxiety, respectively. In the ALV group, 21 patients (30%) presented a depressive state, which required expert consultation, a significantly higher number than in the CLV group (*P* = 0.010) (Table 4). Although a greater number of patients in the ALV group also needed an expert consultation for anxiety, there was no significant difference between the ALV and CLV groups (*P* = 0.076) (Table 4).

**Table 4:** The mean Beck Depression Inventory (BDI) and Beck Anxiety Inventory (BAI) scores, and patients requiring expert consultation based on the results compared between the congenital low-vision and acquired low-vision groups, and between the low-vision and control groups

	Low vision				Control (*n* = 71)	*P* value**
	Congenital (*n* = 55) (%)	Acquired (*n* = 70) (%)	*P* value*	Total (*n* = 125) (%)		
BDI score	7.67 ± 9.04	12.07 ± 11.97	0.021^a^	10.14 ± 10.96	2.77 ± 2.87	<0.001^a^
Patients needing consultation	6 (10.9)	21 (30.0)	0.010^b^	27 (21.6)	0	<0.001^c^
BAI score	5.69 ± 6.85	9.11 ± 10.52	0.030^a^	7.61 ± 9.21	3.55 ± 3.11	<0.001^a^
Patients needing consultation	1 (1.8)	8 (11.4)	0.076^c^	9 (7.2)	0	<0.001^c^

*Comparison between congenital group and acquired group; *P* value < 0.05, statistically significant

**Comparison between low-vision group and control group; *P* value < 0.05, statistically significant

^a^Independent *t*-test

^b^Chi-square test

^c^Fisher’s exact test

To control the confounding effect of age, which is a significant difference between the ALV and CLV groups in demographic data (Table 1), the ANCOVA was performed by settting age as covariate and setting dependent variables as VFQ-25 composite, BAI, and BDI scores. As a result, *P* values of ANCOVA were 0.059, 0.039, and 0.024, respectively. Same as when age was not controlled, there was no statistically significant difference in VFQ-25 composite score between the two groups, and BAI and BDI scores were significantly higher in the ALV group than the CLV group.

**Fig. 1:**
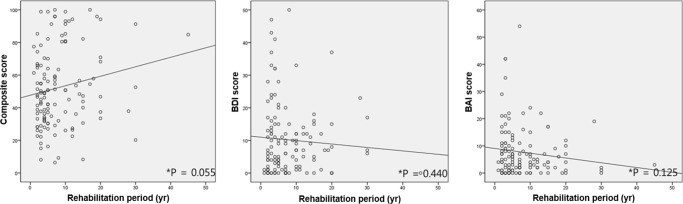
Correlation of the rehabilitation period with the mean composite score for the National Eye Institute Visual Function Questionnaire (NEI VFQ-25) (**a**), the mean score for the Beck Depression Inventory (BDI) (**b**), and the mean score for the Beck Anxiety Inventory (BAI) (**c**) in the low-vision group. There were no significant correlations between the rehabilitation period and questionnaire outcomes. yr year, *Pearson’s correlation coefficient; *P* value < 0.05, statistically significant

In correlation analysis, the rehabilitation duration did not correlate significantly with the composite NEI VFQ-25 score, the BDI score or the BAI score (*P* = 0.055, 0.440, and 0.125, respectively) (Fig. 1).

## Discussion

In this study, the scores for the VR-QoL subscales and the depression and anxiety inventories were significantly worse in low-vision patients than in controls. Subgroup analysis revealed that ALV patients experienced significantly worse depression than CLV patients.

In low-vision rehabilitation, it is important to quantify VR-QoL in order to verify the effects of the intervention. The NEI VFQ-25 is one of the most widely used VR-QoL questionnaires, not only in the ophthalmologic field, but also among normal populations with various languages [18–24]. The NEI VFQ-25 questionnaire was developed as a self-report form; however, due to the deterioration of visual function in low-vision patients, the self-report method can be difficult at times. A previous study found that there was no statistically significant difference in the results from self-reports and interviews [25]. Therefore, in this study, the questionnaire was conducted by either method.

In this cross-sectional study, the mean scores of all the NEI VFQ-25 subscales were lower in the low-vision group than in the control group (Table 2). Previously, Chang et al. compared the NEI VFQ-25 scores among patients with various major ophthalmic diseases, such as low vision, strabismus, diabetic retinopathy, age-related macular degeneration, glaucoma, cataracts, and cytomegalovirus retinitis, and reported that the low-vision group had the lowest scores for most subscales, except for ocular pain [26]. It was obvious that low-vision patients experienced a lower VR-QoL than not only normal controls, but also other patients with ophthalmologic diseases. In addition, the authors hypothesized that the age of onset of low vision would affect the VR-QoL. The subscale and composite score trends of the NEI VFQ-25 were lower in the ALV group than in the CLV group; however, the VR-QoL of low-vision patients was not significantly related to the age of onset of low vision, but rather to degree of visual impairment (Table 3). That is, VR-QoL was principally affected by objective visual function.

In the same context, the mean BDI and BAI scores were significantly higher in the low-vision group than in the normal control group (Table 2). Similar to the subscale and composite score trends for the NEI VFQ-25, the BDI and BAI scores were significantly higher in ALV patients than in CLV patients (Table 4). Thus, the mental health status was poorer in low-vision patients than in normal controls, and was obviously worse in ALV patients than in CLV patients. In particular, 21.6 and 7.2% of low-vision patients needed immediate expert consultation due to depressive or anxiety disorders (Table 4). Also, the proportion of patients requiring depression consultation was significantly higher in the ALV group than in the CLV group (Table 4).

ALV patients usually experience psychological reactions like shock, denial, and depression in the early stage of the disease. Because of these emotional processes, patients must undergo a period of adaptation in order to accept the changes in their life [27]. In an initial adaptation process with impact, time is an important factor that buffers mental stress [28]. However, for patients with an established visual impairment, adaptation can be a continuous process, not a process with a definite endpoint [29]. Patients undergoing this process may be positively influenced by psychological counseling. According to a previous study of an integrated low vision intervention that included mental health counseling with functional training, depression was half as frequent in patients who participated in this intervention than in patients who received traditional supportive therapy [30]. Based on these results, in low-vision rehabilitation, it is important to assess integrated mental health and perform active counseling with interventions, especially for ALV patients.

Interestingly, in correlation analysis, the duration of rehabilitation did not correlate significantly with the composite NEI VFQ-25 score, the BDI score or the BAI score (Fig. 1). We suspected the duration of rehabilitation is other major factor affecting VR-Qol and mental health. However, the result revealed that the duration of rehabilitation was not a major factor that influenced VR-QoL or mental health in low-vision. It seems to require a study of larger subject group.

One limitation of our study is that we did not adjust for possible confounding factors that can affect VR-QoL, such as comorbidities, socioeconomic status, and education level. Unlike previous studies that targeted elderly people, the present study included relatively young subjects. Also, there were only three patients with diabetes and two patients with hypertension in the low-vision group, and no patients with these conditions in the control group. As for the socioeconomic status and education level, because the subjects were reluctant to expose their exact status, the reliability of the data was low. Therefore, we used the area of residence as a proxy of socioeconomic status and education; however, these factors did not differ significantly between groups.

To the best of our knowledge, this is the first report comparing quality of life and mental health through quantitative scales for VR-QoL, depression, and anxiety according to the age of onset of low vision. In conclusion, low-vision patients had lower VR-QoL and mental health states than control subjects, and some of them required expert consultation. The degree of negative impact of low vision was more severe in the ALV group than in the CLV group. Therefore, ophthalmologists and optometrists who participate in low-vision rehabilitation should be aware of the age of onset of low-vision. Furthermore, quantitative evaluation of VR-QoL, depression, and anxiety are needed throughout the rehabilitation process. An integrated rehabilitation approach that not only seeks to improve visual function, but also provides support through psychosocial counseling and intervention is important for optimal outcomes and successful rehabilitation.

## Summary

### What was known before

- In low-vision rehabilitation, visual acuity and visual field tests are objective methods of evaluating visual function. However, such objective examinations cannot be used to measure subjective parameters, such as vision-related quality of life (VR-QoL) and mental health. Also, the impact of the age of onset of low vision on patients’ VR-QoL and mental health has not been studied previously. Thus, the purpose of this study was to evaluate VR-QoL and mental health in low-vision patients and determine the impact of the age of onset by comparing VR-QoL and mental health in patients with congenital low vision and acquired low vision.

### What this study adds

- In low-vision patients had lower vision-related quality of life and mental health states than control subjects, and some of them required expert consultation. The degree of negative impact of low vision was more severe in the acquired low vision group than in the congenital low vision group. Therefore, ophthalmologists and optometrists who participate in low-vision rehabilitation should be aware of the age of onset of low-vision. Furthermore, quantitative evaluation of VR-QoL, depression and anxiety are needed throughout the rehabilitation process. An integrated rehabilitation approach that not only seeks to improve visual function, but also provides support through psychosocial counseling and intervention is important for optimal outcomes and successful rehabilitation.

## Supplementary information

Supplementary table 1
