# Supplementary material for: Comparison of vision-related quality of life and mental health between congenital and acquired low-vision patients
Source: Eye (Lond). 2019 Apr 24;33(10):1540–6. doi: 10.1038/s41433-019-0439-6 (PMC7002576; doi:10.1038/s41433-019-0439-6)
Supplement: Supplementary file 1 — Supplementary table 1 [file 41433_2019_439_MOESM1_ESM.docx]

**Supplementary table 1.** The causes of visual impairment in the congenital low-vision and acquired low-vision groups.

| Diagnosis | Congenital group, n (%) | Acquired group, n (%) | Total, n (%) |
| --- | --- | --- | --- |
| Cornea |  |  |  |
| Corneal opacity | 2 (3.63) | 2 (2.86) | 4 (3.20) |
| Lens |  |  |  |
| Congenital cataract | 4 (7.27) | 0 | 4 (3.20) |
| Retina |  |  |  |
| Macular dystrophy &  degeneration | 9 (16.36) | 20 (28.57) | 29 (23.20) |
| Retinopathy of prematurity | 4 (7.27) | 0 | 4 (3.20) |
| Retinoblastoma | 1 (1.81) | 0 | 1 (0.80) |
| Retinitis pigmentosa | 0 | 9 (12.86) | 9 (7.20) |
| Diabetic retinopathy | 0 | 4 (5.71) | 4 (3.20) |
| Stargardt’s disease | 0 | 3 (4.29) | 3 (2.40) |
| Retinal detachment | 0 | 3 (4.29) | 3 (2.40) |
| Optic nerve |  |  |  |
| Optic atrophy | 18 (32.72) | 14 (20.00) | 32 (25.60) |
| LHON* | 0 | 2 (2.86) | 2 (1.60) |
| Morning glory syndrome | 1 (1.81) | 0 | 1 (0.80) |
| Others |  |  |  |
| Albinism | 7 (12.73) | 0 | 7 (5.60) |
| Nystagmus | 4 (7.27) | 0 | 4 (3.20) |
| Cortical visual impairment | 4 (7.27) | 0 | 4 (3.20) |
| Congenital glaucoma | 1 (1.82) | 0 | 1 (0.80) |
| High myopia | 0 | 6 (8.57) | 6 (4.80) |
| POAG** | 0 | 5 (7.14) | 5 (4.00) |
| Amblyopia | 0 | 2 (2.86) | 2 (1.60) |
| Total | 55 (100) | 70 (100) | 125 (100) |

*LHON = Leber’s hereditary optic neuropathy

**POAG = Primary open angle glaucoma
